# Analysis of the Outcomes Studied in the Application of Invasive and Non-Invasive Vagus Nerve Stimulation in Clinical and Preclinical Studies Involving Stroke—A Scoping Review

**DOI:** 10.3390/neurosci7010009

**Published:** 2026-01-12

**Authors:** Mariana Lara Zambetta, José Mário Prati, Thiago Luiz de Russo, Anna Carolyna Lepesteur Gianlorenço

**Affiliations:** 1 Laboratory of Neuroscience and Neurological Rehabilitation, Physical Therapy Department, Federal University of Sao Carlos, Sao Carlos 13565-905, Brazil; mariana.zambeta@hotmail.com; 2 Department of Physical Therapy, Federal University of Sao Carlos, Sao Carlos 13565-905, Brazil; russo@ufscar.br; 3 Spaulding Neuromodulation Center, Harvard Medical School, Cambridge, MA 02138, USA

**Keywords:** stroke, invasive vagus nerve stimulation, non-invasive vagus nerve stimulation

## Abstract

Background: Currently, there is a considerable number of studies addressing vagus nerve stimulation (VNS) for the treatment of different stroke-related outcomes. We aimed to promote a broad view of the outcomes studied and what are the opportune outcomes to be studied involving this therapeutic strategy for the treatment of post-stroke complications. Methods: This is a scoping review that followed the Preferred Reporting Items for Systematic Reviews and Meta-Analyses (PRISMA). Two investigators conducted independent searches on PubMed/MEDLINE, Scopus, and Embase till July 2025. Randomized clinical trials and preclinical studies using invasive or non-invasive vagus nerve stimulation conducted with a population diagnosed with stroke were included. Results: Forty-one experimental studies and sixteen clinical trials were included. The outcomes found were neuroprotection; motor, functional, and cognitive rehabilitation; dysphagia; comparison of different stimulation intensities; safety, efficacy, and feasibility of the non-invasive approach; comparison between transcutaneous auricular vagus nerve stimulation (taVNS) and transcutaneous cervical vagus nerve stimulation (tcVNS); and comparison between two models of ischemia (permanent and transient). Preclinical studies mostly investigated molecular elements involved in neuroprotection, neuroinflammation, and cellular apoptosis, while clinical studies evaluating the effectiveness of this technique used for rehabilitation and its comparison or combination with other techniques remain scarce. Conclusions: Most studies investigating the effects of VNS on different post-stroke outcomes are experimental studies. Clinical studies are still scarce and with limited analysis of outcomes.

## 1. Introduction

Stroke is a cerebrovascular disease [1] that affects millions of people worldwide annually and can be classified as ischemic stroke, which results from the obstruction of a blood vessel by a clot or atherosclerotic plaque, or hemorrhagic stroke, caused by the rupture of a blood vessel and the leakage of blood into the nervous tissue [2].

Stroke incidence is higher in women (48%) and in people under 70 years old (almost 60%). However, mortality and disability rates are higher in men (53%) [3]. On the other hand, the overall prevalence of stroke in older adults is 7.4% and increases with age [4]. The main risk factors include high systolic blood pressure, atrial fibrillation, an unbalanced diet, a high body mass index, and elevated fasting glucose levels [5]. A recent study demonstrated that the costs of caring for stroke patients—including the acute post-stroke phase and rehabilitation—range from GBP 610.00 to GBP 220,822.45 per patient annually. Additionally, the study showed that post-stroke care costs (49.4%) are higher than stroke prevention care costs (8.8%) [6].

Stroke also has a major direct impact on the functionality and quality of life of patients, commonly leading to short- and long-term disabilities, such as sensory disorders, cognitive disorders, speech disorders, hemiplegia, decline in self-care ability, emotional and socioeconomic consequences, among others [7].

Over the years, several therapeutic resources have been developed to treat different outcomes, aiming to improve the functional independence of individuals affected by stroke. Currently, the most widely used treatment methods for rehabilitation are techniques based on intensive, task-specific, and repetitive interventions [8]. However, neuromodulation techniques, such as transcranial magnetic stimulation (TMS), transcranial direct current stimulation (tDCS), and vagus nerve stimulation (VNS), have rapidly gained ground in neurological rehabilitation, both as a treatment modality to mitigate the deleterious effects of stroke and to promote neuroplasticity and recovery in chronic stroke [9].

The vagus nerve conducts visceral and somatic afferent information to the nucleus of the solitary tract (NTS). The NTS, in turn, projects to several other neuronal nuclei, including the ambiguous nucleus, the dorsal motor nucleus of the vagus nerve, the parabrachial nucleus, the hypoglossal nucleus, the facial nucleus, the spinal trigeminal nucleus, the locus coeruleus, the Raphe nuclei, the periaqueductal gray matter, and the Kölliker–Fuse nucleus [10].

VNS has been shown to produce an anti-inflammatory effect through the cholinergic anti-inflammatory pathway, promoting the release of acetylcholine and activation of the α7nAChR receptor in immune system cells. Additionally, VNS regulates the release of neurotransmitters, including gamma-aminobutyric acid (GABA), noradrenaline, serotonin, and dopamine in brain regions such as the locus coeruleus, basolateral amygdala, ventral tegmental area, hippocampus, and prefrontal cortex. VNS promotes neuroplasticity through the brain-derived neurotrophic factor (BDNF)—Tropomyosin receptor kinase B (TrkB) pathway, and promotes reduction in cell apoptosis and autophagy through caspase-3 inhibition, increased miR-210 expression, and downregulated expression of autophagy-related proteins Beclin-1 and LC3-II. Finally, VNS promotes a protective effect on the blood–brain barrier and enhances angiogenesis through the expression of BDNF, vascular endothelial growth factor (VEGF), growth differentiation factor 11 (GDF-11), and endothelial nitric oxide synthase (eNOS) [11].

Invasive vagus nerve stimulation (iVNS) and non-invasive VNS have shown promising results in upper limb function, especially. When paired with rehabilitation exercises, iVNS has shown potential in enhancing neuroplasticity and improving motor function recovery in stroke survivors [12]. It is a therapeutic strategy approved by the Food and Drug Administration (FDA) in 2005 [13], which delivers electrical signals to the vagus nerve. This method involves surgically implanting a device to deliver electrical impulses to the cervical branch of the vagus nerve. Due to its surgical nature, it carries associated risks and higher costs compared to non-invasive alternatives like transcutaneous auricular VNS (taVNS) or transcutaneous cervical VNS (tcVNS), targeting, respectively, the auricular or cervical branch of the vagus nerve [14].

It has been shown that stimulation of the cymba conchae and tragus promoted increased activity in the bilateral NTS, bilateral locus coeruleus, right thalamus, putamen, caudate, bilateral corpus callosum, frontal and central operculum, right anterior insula, and cerebellum [15]. Additionally, in another study, tragus stimulation produced increased activity in the cerebellum, bilateral anterior and middle cingulate cortex, left prefrontal cortex, and right caudate compared to earlobe stimulation [16]. These findings demonstrate that stimulation of the tragus and cymba conchae serves as a pathway for stimulating the auricular branch of the vagus nerve and other brain structures that may be affected by the occurrence of a stroke. The increased activity of these structures through auricular vagus nerve stimulation may be reflected in improvements in clinical outcomes related to stroke, such as upper limb function, speech, swallowing, and other outcomes.

In summary, stroke causes a great burden to affected individuals and their families, and despite the great development of treatment techniques in recent years, rehabilitation remains limited in many cases, highlighting the importance of continued efforts to better understand and develop both pathophysiological mechanisms and treatment approaches. VNS has been a major focus of research in stroke conditions, both in basic research and in clinical trials. Therefore, the aim of this study is to conduct a scoping review to identify the main outcomes analyzed in preclinical and clinical studies in the literature that have studied the effects of VNS for the treatment of stroke-related complications. This review aims to contribute to the guidance of new clinical and preclinical studies that use VNS as a resource in the treatment of stroke, based on outcomes that have not yet been studied or have been little investigated in this specific context.

## 2. Materials and Methods

### 2.1. Search Strategy

This is a scoping review following the Preferred Reporting Items for Systematic Reviews and Meta-Analyses (PRISMA) guidelines. This review was previously registered in the Open Science Framework (OSF) under the DOI 10.17605/OSF.IO/AGS8X. To achieve the objective of this study, a comprehensive search strategy was employed in the PubMed, Scopus, and Embase databases using the search strategy: “vagus nerve stimulation” OR “transcutaneous vagus nerve stimulation,” AND “stroke”. In a prior analysis of the literature, it was observed that there was compatibility between the defined search terms and the studies available in the databases. Therefore, the outlined search strategy was designed objectively, aiming to capture the maximum number of studies related to the research topic. The searches began in June 2025 and were conducted until July 2025.

### 2.2. Eligibility Criteria

The inclusion criteria established were (i) clinical trials using invasive or non-invasive vagus nerve stimulation conducted on a population diagnosed with stroke; (ii) studies with at least one control group; (iii) experimental studies with animal models of stroke; (iv) studies published in English; (v) studies available in full text. There were no publication date limitations for the inclusion of studies. Review studies, protocols, observational studies, case reports, conference proceedings, and posters were not considered eligible.

### 2.3. Data Selection and Extraction Procedure

The studies retrieved from the databases were exported to the Rayyan reference manager [17]. During the screening process, duplicates were removed, and study selection was performed independently by two researchers. The studies included after this initial analysis were read in full. Conflicts were resolved through discussion and consensus among the researchers. Those that met the inclusion criteria were tabulated, and their data were extracted. This review has an exploratory nature; therefore, no statistical tests were required based on the presented results.

## 3. Results

**Figure 1:**
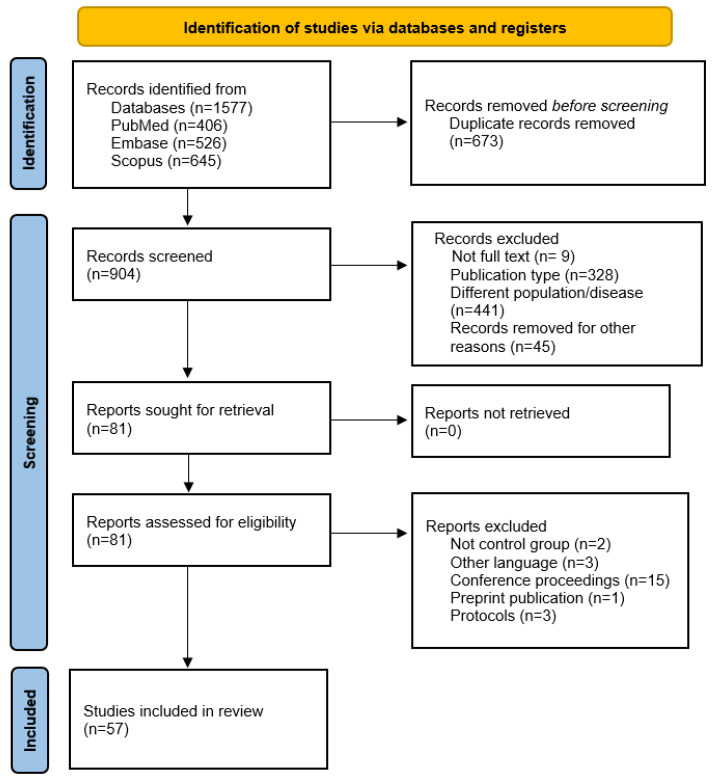
PRISMA-ScR flow diagram illustrating the systematic search across the databases: preclinical studies and clinical trials included in the review.

Our electronic searches returned 1577 studies. After the article selection process and application of the eligibility criteria, 57 studies were included in the review. The selection process, including the reasons for exclusion of records, is described in Figure 1.

### 3.1. Overall Characteristics of the Experimental Studies

**Figure 2:**
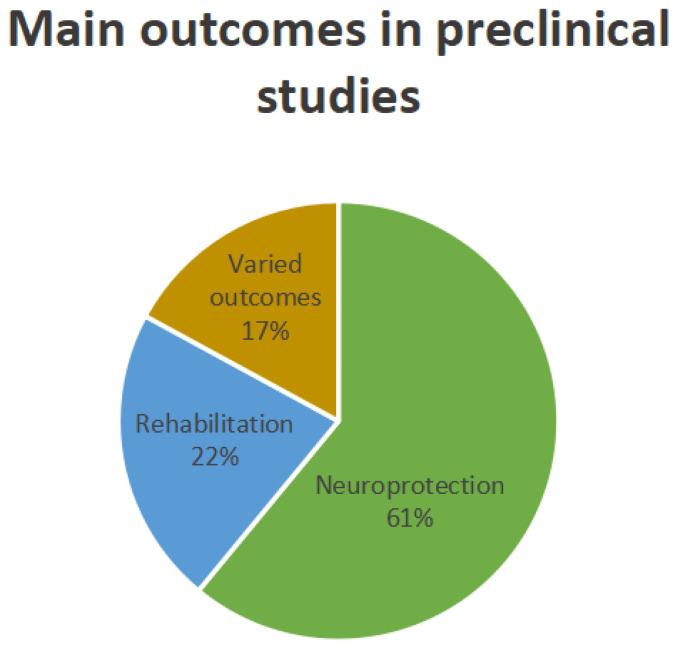
Graphical representation of the main outcomes found in preclinical studies: outcomes for neuroprotection (61%), outcomes for rehabilitation (22%), and varied outcomes (17%).

Of the 57 studies included, 41 were preclinical studies in rodent models. A descriptive characterization of the preclinical studies is summarized in Table 1. Twenty-five studies investigated the effects of VNS-induced neuroprotection (61%), nine studies investigated the effects of VNS on motor, functional or cognitive rehabilitation (22%), and seven studies presented mixed outcomes (17%) (Figure 2), such as verifying the effects of taVNS on cardiac dysfunction [18] and effects on mast cell degranulation related to myocardial atrophy after acute stroke [19]; effects of taVNS on dysphagia symptoms after ischemic stroke [20]; comparison of different intensities of VNS for motor rehabilitation [21]; safety and efficacy of the non-invasive approach of vagus nerve stimulation (nVNS) [22]; comparison of the effects of applying auricular vagus nerve stimulation and cervical vagus nerve stimulation and whether this was associated with a reduction in infarct volume and improvement in functional outcomes [23]; and investigation of neuroprotection of VNS comparing two models of ischemia: transitory proximal middle cerebral artery occlusion (tMCAO) and permanent middle cerebral artery occlusion (pMCAO) [24]. Most studies have applied VNS in the hyperacute phase of stroke.

Of the forty-one animal model studies, twenty-six used Sprague–Dawley mice, five studies used C57BL/6 mice, four studies used Wistar rats, and six studies did not specify the rodent strain. Most studies applied iVNS, totaling twenty-six studies [21,22,23,24,25,26,27,28,29,30,31,32,33,34,35,36,37,38,39,40,41,42,43,44,45,46]; ten studies applied taVNS [18,20,47,48,49,50,51,52,53,54]; four studies applied tcVNS [19,55,56,57]; and one study applied iVNS and tcVNS [58].

**Figure 3:**
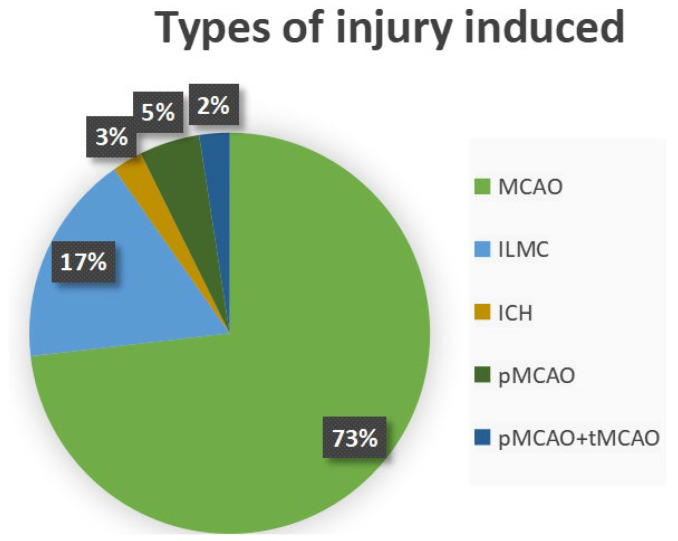
Graphical representation of stroke models found in preclinical studies: MCAO: middle cerebral artery obstruction (73%), ILMC: ischemic lesion in the motor cortex (17%), ICH: intracerebral hemorrhage (3%), pMCAO: permanent middle cerebral artery obstruction (5%), comparison between pMCAO and tMCAO: temporary proximal middle cerebral artery occlusion (2%).

Furthermore, with regard to type of injury models for induction of vascular impairment, thirty studies performed middle cerebral artery occlusion (MCAO) (73%), seven studies performed ischemic lesion in the motor cortex (ILMC) (17%), two studies used pMCAO (5%), one study performed spontaneous intracerebral hemorrhage (ICH) (3%), and one study compared pMCAO with tMCAO (2%) (Figure 3).

In relation to the parameters applied, the frequency varied between 2 and 30 Hz, and the stimulation intensity varied between 0.2 mA and 2.0 mA. Eighteen studies investigated the acute effects of stimulation, applying it once with a total duration of 1 h [19,20,21,22,23,24,25,26,29,30,33,34,35,47,49,50,57,58]. One study applied stimulation for 30 min daily for 3 days [18]; one study applied stimulation for 1 h daily for 14 or 28 days, depending on group allocation [27]; one study applied stimulation for 20 min daily for 3 days [28]; two studies applied stimulation once for 45 min [55,56]; one study applied stimulation for 30 min daily for 5 days [53] and 7 days [48]; one study applied stimulation for 10 min daily for 5 days [31]; one study applied stimulation once for 30 min [32]; one study applied stimulation for 1 h daily for 21 days [51], 5 days [52], and 3 days [37]; one study applied stimulation once for 10 min [36]; one study applied stimulation for 30 min daily for 7 days [38]; two studies applied stimulation for 25 days [40,43]; four studies applied stimulation for 5 weeks [39,44,45,46]; one study applied stimulation for 6 weeks [41]; and one study applied stimulation for 30 min daily for 3 weeks [54].

Most preclinical studies have demonstrated positive effects of VNS in the treatment of various stroke-related outcomes. Only one study reported non-significant results, showing that the infarct-reducing effect provided by VNS was not mediated by an increase in cerebral blood flow [33].

**Table 1:** Characterization of preclinical studies included in the review.

Author/Year	Injury/Sample	Time-Point of Intervention	Intervention	Outcomes/Results
taVNS				
Wang et al., 2025 [18]	MCAO *n* = 48 male C57BL/6N	Hyperacute stroke	Intensity: 0.2 mA Frequency: 2/15 Hz Duration: 30 min daily for 3 days	To verify the effects of taVNS on cardiac dysfunction. Result: significant.
Ay et al., 2015 [20]	MCAO *n* = 31 males Wistar	Hyperacute stroke	Intensity: 0.5 mA Frequency: 20 Hz Duration: 1 h	To determine whether taVNS activated the same vagal nuclei activated by the gold standard cVNS and whether this was associated with a reduction in infarct volume and improvement in functional outcomes. Result: significant.
Li et al., 2020 [47]	MCAO *n* = 64 male SD	Hyperacute stroke	Intensity: 0.5 mA Frequency: 20 Hz Duration: 1 h	To investigate the effects of taVNS on axonal plasticity through the activation of α7nAChR in animal models of stroke. Results: significant.
Zhao et al., 2022 [48]	MCAO *n* = 48 male SD	Hyperacute stroke	Intensity: 1.0 mA Frequency: 10 Hz Duration: 30 min daily for 7 days	To evaluate the effects of taVNS on inflammation in the ischemic penumbra and motor cortex, expression and phosphorylation of Cx43, and neurological function in animal models of stroke. Result: significant.
Li et al., 2020 [49]	MCAO *n* = 72 males SD	Hyperacute stroke	Intensity: 0.5 mA Frequency: 20 Hz Duration: 1 h	To investigate whether PPAR-γ was involved in the pro-angiogenic activity induced by taVNS and its mechanism after ischemic brain injury. Results: significant.
Ma et al., 2016 [50]	MCAO *n* = 218 males SD	Hyperacute stroke	Intensity: 0.5 mA Frequency: 20 Hz Duration: 1 h	To investigate the effects of taVNS on the expression of GDF11 in the spleen, plasma, and peri-infarct cerebral cortex, and its receptor ALK5. Results: significant.
Jiang et al., 2016 [51]	MCAO *n* = 224 males SD	Hyperacute stroke	Intensity: 0.5 mA Frequency: 20 Hz Duration: 1 h daily for 21 days	To investigate the effects of taVNS on functional recovery, neovascularization, and the expression of pro-angiogenic mediators. Results: significant.
Wu et al., 2018 [52]	MCAO *n* = 70 males SD	Acute stroke	Intensity: 0.8 mA Frequency: 15 Hz Duration: 1 h daily for 5 days	To verify the effects of taVNS on cognitive impairment resulting from stroke and the role of the non-neuronal cholinergic system in the management of acetylcholine release and reuptake. Results: significant.
Gong et al., 2025 [53]	MCAO *n* = 10 male C57BL/6J	Acute stroke	Intensity: 1.0 mA Frequency: 20 Hz Duration: 30 min for 5 days	To investigate the effects of taVNS on the temporal dynamics and mitigation of ferroptosis and neurological recovery. Results: significant.
Long et al., 2022 [54]	MCAO *n* = 65 SD	Not specified	Intensity: 2.0 mA Frequency: 20 Hz Duration: 30 min daily for 3 weeks	To investigate the effects of taVNS on dysphagia symptoms and white matter damage in animals with dysphagia after ischemic stroke. Results: significant.
tcVNS				
Ay et al., 2016 [19]	MCAO *n* = 54 males	Hyperacute stroke in spontaneously hypertensive rats	1 msec duration, 5 kHz, 12 V sine waves repeated at 25 Hz. Duration: 1 h.	To explore the safety and efficacy of a non-invasive tcVNS approach using surface electrodes applied to the skin overlying the vagus nerve in the neck in a model of MCAO in rats. Result: significant.
Yang et al., 2022 [55]	tMCAO *n* = 32 males	Hyperacute stroke in spontaneously hypertensive rats	1 ms pulses width of 5 kHz sinewaves, repeated at 25 Hz, at an average voltage of 15 V. Duration: 45 min.	To verify the hypothesis that nVNS reduced neuron-derived IL-1β and neuroinflammation in acute ischemia, using a SHR model of MCAO/RP. Result: significant.
Yang et al., 2018 [56]	MCAO *n* = 32 males	Hyperacute stroke in spontaneously hypertensive rats	1 msec pulses width of 5 kHz sinewaves, repeated at 25 Hz, at an average voltage of 15 V. Duration: 45 min.	To investigate the effects of nVNS on the reduction in ischemic infarct size, associated with the protection of the blood–brain barrier. Result: significant.
Zhao et al., 2019 [57]	MCAO *n* = 20 males C57BL/6	24 h before MCAO	1 ms pulses, width of 5 kHz sinewaves, repeated at 25 Hz, with an average voltage of 15 V Duration: 1 h.	To investigate the effects of VNS on reducing ischemia/reperfusion-induced injury and the M2 action of microglia through the inhibition of IL-17A expression. Results: significant.
iVNS				
Sun et al., 2012 [21]	tMCAO/pMCAO *n* = 32 males SD	Hyperacute stroke	Intensity: 0.5 mA Frequency: 20 Hz Duration: 1 h	To examine the effects of VNS on cerebral infarct volume and neuroprotection in animals exposed to pMCAO and tMCAO. Result: significant.
Liu et al., 2023 [22]	MCAO *n* = 96 males SD	Hyperacute stroke	Intensity: 0.5 mA Frequency: 20 Hz Duration: 1 h	To investigate whether VNS is a new potential therapeutic option for ischemic stroke and whether α7nAChR is associated with the VNS-mediated shift in the microglial phenotype after ischemic brain injury. Result: significant.
Tang et al., 2022 [23]	MCAO *n* = 23 males SD	Hyperacute stroke	Intensity: 0.5 mA Frequency: 20 Hz Duration: 1 h	To verify the influence of α7nAChR on mediating the neuroprotection induced by VNS in inhibiting pyroptosis in ischemia–reperfusion brain injury. Result: significant.
Lu et al., 2017 [24]	pMCAO *n* = 108 males SD	Hyperacute stroke	Intensity: 0.5 mA Frequency: 20 Hz Duration: 1 h	To investigate the effects of VNS on neuroprotection by evaluating neurological function and infarct volume, and identify whether α7nAChR plays a role in VNS-mediated neuroprotection. Result: significant.
Jiang et al., 2015 [25]	MCAO *n* = 360 males SD	Hyperacute stroke	Intensity: 0.5 mA Frequency: 20 Hz Duration: 1 h	To examine the stimulation efficiency of VNS by measuring the expression of α7nAChR in neurons and astrocytes, and by determining neurological scores, infarct volume, and neuronal apoptosis. Also, to explore the molecular effects of miR-210 in the VNS response by assaying the levels of three oxidative stress markers and caspase 3 activity in ischemic stroke. Result: significant.
Tang et al., 2025 [26]	MCAO *n* = male SD	Hyperacute stroke	Intensity: 0.5 mA Frequency: 20 Hz Duration: 1 h	To verify the effects of VNS on the regulation of PANoptosis through Sirt1. Result: significant
Jiang et al. 2024 [27]	MCAO *n* = male C57BL/6	Hyperacute stroke	Intensity: 0.5 mA Frequency: 20 Hz Duration: 1 h daily for 3 or 28 days	To evaluate the role of VNS in the secretion of Neuromedin U and its receptors on astrocytes and the regulation of A1/A2 polarization of astrocytes. Result: significant.
Wang et al. 2024 [28]	MCAO *n* = male SD	Hyperacute stroke	Intensity: adjusted Frequency: 15 Hz Duration: 20 min daily for 3 days	To investigate whether VNS can modulate mast cell degranulation via α7nAChRs or other pathways, causing damage to the blood–brain barrier and intestinal barrier. Result: significant.
Zhang et al., 2021 [29]	MCAO *n* = 150 males SD	Hyperacute stroke	Intensity: 0.5 mA Frequency: 20 Hz Duration: 1 h	To evaluate the effects of VNS on microglial polarization through the inhibition of the TLR4 pathway in microglia in ischemic stroke injury. Result: significant.
Zhang et al., 2016 [30]	MCAO *n* = 272 animals SD	Hyperacute stroke	Intensity: 0.5 mA Frequency: 20 Hz Duration: 1 h	To investigate the function of L-PGDS and its involvement in the anti-apoptotic activity induced by VNS. Result: significant.
Du et al., 2022 [31]	MCAO *n* = 80 males C57BL/6J	Hyperacute stroke	Intensity: 1.0 mA Frequency: 5 Hz Duration: 10 min daily for 5 days	To evaluate the protective effect of VNS on cerebral ischemic injury by analyzing changes in proteins and signaling pathways. Result: significant.
Ekıcı et al., 2013 [32]	MCAO *n* = 21 males Wistar	Hyperacute stroke	Intensity: 1.0 mA Frequency: 20 Hz Duration: 30 min	To investigate the effects of VNS in ischemia–reperfusion injury by measuring infarct area and neurological scores, and to analyze oxidative stress markers. Result: significant.
Ay et al., 2011 [33]	MCAO *n* = 32 males Wistar	Hyperacute stroke	Intensity: 0.5 mA Frequency: 20 Hz Duration: 1 h	To explore whether infarct-reducing effect of VNS is mediated by an increase in cerebral blood flow. Result: non-significant.
Lindemann et al., 2020 [58]	pMCAO *n* = male Wistar	Hyperacute stroke	Intensity: 0.5 mA Frequency: 25 Hz Duration: 1 h	To determine the effects of VNS (invasive and non-invasive) on inhibiting spreading depolarization in animal models of focal ischemia. Result: significant.
Hiraki et al., 2012 [34]	MCAO *n* = 28 males SD	Hyperacute stroke	Intensity: 0.5 mA Frequency: 20 Hz Duration: 1 h	To investigate the effect of VNS on infarct volume and neurological recovery up to three weeks following transient focal cerebral ischemia. Results: significant.
Jiang et al., 2015 [35]	MCAO *n* = 160 males SD	Hyperacute stroke	Intensity: 0.5 mA Frequency: 20 Hz Duration: 1 h	To investigate the role of endogenous PPAR-γ in anti-inflammatory actions induced by VNS during reperfusion after stroke, a mechanism thought to reduce neuronal injury in the brain. Results: significant.
Liu et al., 2016 [36]	MCAO *n* = 34 males SD	Hyperacute stroke	Intensity: 1.0 mA Frequency: 20 Hz Duration: 10 min	To analyze the contribution of VNS in the recovery of learning and memory after I/R injury and the involved mechanisms. Results: significant.
Xie et al., 2023 [37]	MCAO *n* = 200 mice	Hyperacute/acute stroke	Intensity: 0.5 mA Frequency: 5 Hz Duration: 1 h for 3 days	To hypothesize that VNS achieves cerebral protection by influencing NF-kB-related neuroinflammation via USP10 regulation. To clarify the protective effects of VNS following ischemic stroke and explore the underlying mechanisms. Result: significant.
Tan et al. 2024 [38]	MCAO *n* = 60 SPF-grade male SD	Acute stroke	Intensity: adjusted Frequency: 15 Hz Duration: 30 min daily for 7 days	To analyze the effects of VNS on myocardial atrophy. Results: significant.
Hays et al., 2016 [39]	ILMC *n* = 344 rats	Acute stroke	Intensity: 0.8 mA Frequency: 30 Hz Duration: 5 weeks	To evaluate the effects of VNS paired with rehabilitation training on improving forelimb function in animals with ischemic injury aged at least 18 months. Results: significant.
Khodaparast et al. 2014 [40]	ILMC *n* = 17 female SD	Acute stroke	Intensity: 0.8 mA Frequency: 30 Hz Duration: 25 days	To evaluate whether the addition of VNS to motor rehabilitation can enhance recovery from cortical ischemia. Results: significant.
Meyers et al., 2018 [41]	ILMC *n* = 19 females SD	Acute stroke	Intensity: 0.8 mA Frequency: 30 Hz Duration: 6 weeks	To test whether ENV could promote generalization, lasting recovery, and structural plasticity in motor networks. Results: significant.
Khodaparast et al., 2013 [42]	ILMC *n* = 19 females SD	Acute stroke	Intensity: 0.8 mA Frequency: 30 Hz Duration: Fifty sessions, 30 min each session	To evaluate whether the delivery of VNS during rehabilitative training can enhance recovery of forelimb strength in a model of ischemic stroke. Results: significant.
Hays et al., 2014 [43]	ILMC *n* = 32 females SD	Acute stroke	Intensity: 0.8 mA Frequency: 30 Hz Duration: 25 days	To test the effectiveness of different stimulation paradigms to restore forelimb strength after ischemic lesion of the motor cortex in rats. Results: significant.
Hays et al., 2014 [44]	ICH *n* = 26 females SD	Acute stroke	Intensity: 0.8 mA Frequency: 30 Hz Duration: 5 weeks	To evaluate whether VNS paired with rehabilitative training can improve recovery of motor function beyond rehabilitative training without VNS in a rat model of ICH. Result: significant.
Pruitt et al. 2020 [46]	ILMC *n* = 32 females SD	Acute stroke	Intensity: 0.4, 0.8, and 1.6 mA Frequency: 30 Hz Duration: 5 weeks	To investigate the stimulation intensity to optimize motor function recovery in an animal model of ischemic stroke. Result: significant for moderate intensity.
Khodaparast et al., 2016 [45]	ILMC *n* = 29 females SD	Chronic stroke	Intensity: 0.8 mA Frequency: 30 Hz Duration: 5 weeks	To determine whether VNS-paired rehabilitative training enhances recovery of forelimb function when the therapy is initiated during the chronic phase after a combined cortical and subcortical ischemic stroke. Results: significant.

MCAO: middle cerebral artery occlusion; SD: Sprague–Dawley; VNS: vagus nerve stimulation; taVNS: transcutaneous auricular vagus nerve stimulation; SPF: Specific Pathogen-Free; ILMC: ischemic lesion in the motor cortex; tcVNS: transcutaneous cervical vagus nerve stimulation; iVNS: invasive vagus nerve stimulation; tMCAO: transient middle cerebral artery occlusion; pMCAO: permanent middle cerebral artery occlusion; α7nAChR: α7 nicotinic acetylcholine receptor; miR-210: microRNA-210; Sirt1: sirtuin 1; NF-kB: transcription factor nuclear factor-kB; USP10: ubiquitin-specific protease 10; IL-1β: interleukin-1 beta; SHR: spontaneously hypertensive rat; RP: reperfusion; Cx43: Cnnexin 43; TLR4: Toll-like receptors; IL-17A: interleukin-17A; L-PGDS: lipocalin prostaglandin D2 synthase; PPAR-γ: peroxisome proliferator-activated receptor γ; GDF11: growth differentiation factor 11; ALK5: activin-like kinase 5; I/R: ischemia/reperfusion; ICH: intracerebral hemorrhage.

### 3.2. Effects of VNS on Neuroprotection After Stroke

#### 3.2.1. Nicotinic Acetylcholine Receptor (α7nAChR)

Six studies have investigated the neuroprotective effects from outcomes involving the activation of the VNS α7nAChR after stroke. The outcomes focused on investigating the therapeutic effects of taVNS in regulating ferroptosis through the involvement of the α7nAChR receptor as a mediating mechanism [53], and whether α7nAChR could alter the microglial phenotype [22] and could act in the mediation of induced neuroprotection [23,24]. The effects of axonal plasticity by activation of α7nAChR were also investigated, leading to positive regulation of BDNF-associated signaling [47]. Furthermore, α7nAChR expression in neurons and astrocytes was measured, as well as the influence of α7nAChR on neurological scores, infarction volume, and neuronal apoptosis, and on the molecular effects of MIR-210 on the VNS response, evaluating oxidative stress markers and caspase activity 3 [25].

#### 3.2.2. Neuroinflammatory Mechanisms

Ten studies investigated neuroprotective effects based on outcomes involving stroke-induced neuroinflammatory mechanisms. The outcomes included the effects of VNS on PANoptosis through the expression of Sirt1 [26]; the effects of VNS on Neuromedin U cartilage and expression of its receptors on the regulation of polarization of A1/A2 types of astrocytes [27]; promotion of mast cell degranulation by VNS, impacting blood–brain barrier and intestinal barrier damage [28]; inhibition of the activation of the transcription factor nuclear factor-κB (NF-κB) through the neutralization of USP10 by the application of VNS [37]; reduction in neuron-derived interleukin-1 beta (IL-1β) and neuroinflammation in acute ischemia using a spontaneously hypertensive rat model [55]; inflammatory responses of Cx43 activation, which may mediate a nociceptive effect by propagating brain damage [48]; microglial polarization through inhibition of the Toll-like receptor (TLR4) pathway in microglia [29]; M2 microglial polarization by inhibiting the expression of interleukin-17A (IL-17A) [57]; and the role of Lipocalin prostaglandin D2 synthase (L-PGDS) in the antiapoptotic activity induced by VNS after MCAO [30]; and another study evaluated the protective effect of VNS post-MCAO through changes in proteins and signaling pathways for cell apoptosis and regulation of autophagy [31].

#### 3.2.3. Cerebral Infarct Volume

Five studies were found that investigated the neuroprotective effects of VNS based on outcomes involving a reduction in cerebral infarct volume after MCAO. The observed outcomes included the spatiotemporal correlation of blood–brain barrier (BBB) protection [56]; neurological scores and analysis of oxidative stress markers [32]; the effects of VNS on infarct reduction mediated by increased cerebral blood flow (CBF) [33]; inhibition of spreading depolarization (SD) [58]; and the effects of VNS on infarct volume and neurological recovery up to three weeks after MCAO [34]. The cerebral infarct volume after MCAO and treatment with VNS was investigated using magnetic resonance imaging [56], performing 2,3,5-triphenyltetrazolium chloride (TTC) labeling [32,33,58], and performing delineation of the injured brain area using a computer-based image analyzer [34].

#### 3.2.4. Pro-Angiogenesis

Four studies investigated the neuroprotective effects of VNS based on outcomes involving pro-angiogenic activity. Outcomes included involvement of peroxisome proliferator-activated receptor γ (PPAR-γ) [49], endogenous role of PPAR-γ in VNS-induced anti-inflammatory actions during reperfusion after transient MCAO [35], expression of growth differentiation factor (GDF11) in the spleen, plasma, and peri-infarct cortex and its receptor activin-like kinase 5 (ALK5) [50], and functional recovery, neovascularization, and expression of some pro-angiogenic mediators [51].

### 3.3. Effects of VNS on Motor, Functional, or Cognitive Rehabilitation

Two studies investigated the effects of VNS combined with rehabilitation in an animal model submitted to MCAO. The outcomes included cognitive recovery through the reuptake and release of acetylcholine by the non-neuronal cholinergic system (NNCS) after transcutaneous vagus nerve stimulation (tVNS) [52] and recovery of learning, memory, and the mechanisms involved [36].

Seven studies evaluated the effects of VNS combined with rehabilitation in animal models submitted to primary motor cortex injury. The main outcomes, in turn, were functional recovery [39,40]; functional recovery for untrained tasks, durability of improved functional recovery, and neuroplasticity in the corticospinal tract by retrograde transneuronal tracing [41]; functional recovery of the forelimb with therapy initiated in the chronic and acute phases [42,45]; and forelimb strength [43]. Finally, a study evaluated the recovery of motor function in an animal model of spontaneous intracerebral hemorrhage [44].

### 3.4. Overall Characteristics of the Clinical Studies

The descriptive characterization of the 16 clinical studies is summarized in Table 2, and the clinical characterization of the clinical trial participants is in Table 3. A total of 987 people with stroke participated in the studies, of which 480 were women (49%), and 507 were men (51%), showing a homogeneous sample. The age of study participants ranged from 45 to 79 years old.

**Figure 4:**
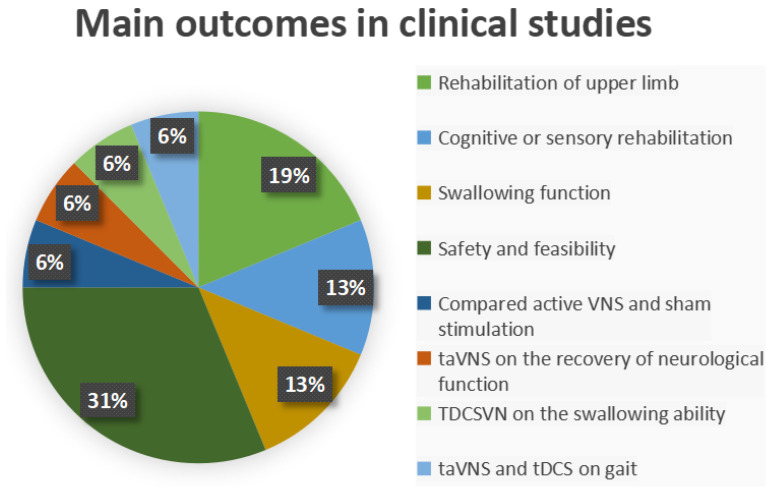
Graphical representation of the main outcomes found in clinical studies: outcomes for rehabilitation of upper limb (19%), outcomes for cognitive or sensory rehabilitation (13%), outcomes for swallowing function (13%), outcomes for safety and feasibility (31%), outcomes for compared active and sham VNS (6%), outcomes for taVNS on the recovery of neurological function (6%), TDCSVN on the swallowing ability (6%), and outcomes for taVNS and tDCS on gait (6%).

**Figure 5:**
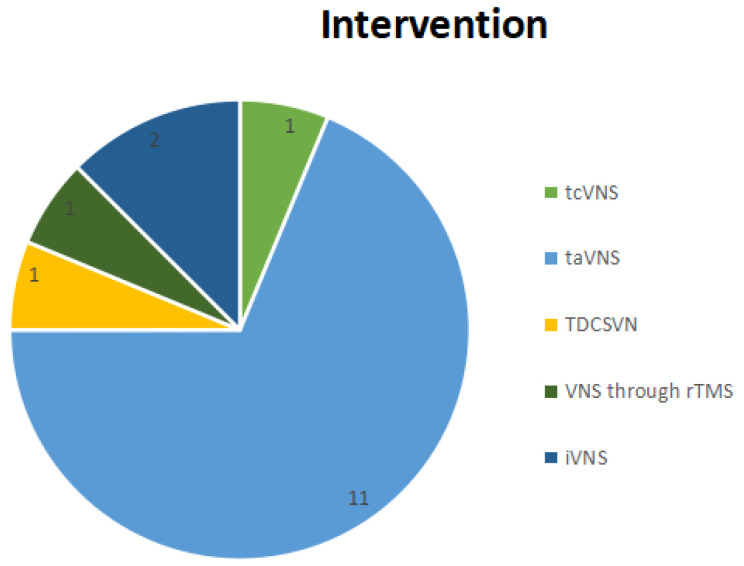
Graphical representation of the intervention found in clinical studies: tcVNS (1), taVNS (11), TDCSVN (1), VNS through rTMS (1), and iVNS (2).

Three studies investigated the effects of VNS on the rehabilitation of upper limb motor function [59,60,61]; one study analyzed the effects of taVNS on the recovery of neurological function and remodeling of the central nervous system [62]; two studies assessed the effects of taVNS on cognitive or sensory rehabilitation and emotional responses [63,64]; two studies investigated the effects of VNS on swallowing function [65,66] and one study evaluated the effects of transcranial direct current stimulation of the vagus nerve on the swallowing ability of individuals after stroke [67]; four studies evaluated the safety and/or feasibility of VNS application in patients after stroke [68,69,70,71]; one study for taVNS post-stroke depression [72]; one study compared active VNS paired with rehabilitation versus sham stimulation paired with rehabilitation in people with moderate to severe arm impairment after ischemic stroke [73]; and finally, only one study investigated the effects of taVNS and tDCS on gait in individuals after subacute stroke [74] (Figure 4). Twelve studies involved individuals with either ischemic or hemorrhagic stroke [59,60,61,62,63,64,65,66,67,68,72,74], and four studies involved only individuals with ischemic stroke [69,70,71,73]. One study applied VNS during the hyperacute phase of stroke [68], eight studies applied stimulation during the subacute phase of stroke [59,62,64,66,67,69,72,74], six studies applied stimulation during the chronic phase of stroke [60,61,65,70,71,73], and one study did not specify the phase of stroke in which stimulation was applied [63]. Eleven studies applied taVNS [59,60,61,62,63,64,66,67,69,72,74], two studies applied iVNS [71,73], one study applied taVNS and tDCS [74], one study applied tcVNS [68], one study applied TDCSVN [70], and one study applied VNS through rTMS [65], as shown in Figure 5.

The characterization of the parameters used in the clinical trials is summarized in Table 4. Regarding the stimulation parameters, the intensity was adjusted according to the patient’s tolerance in seven studies [61,62,65,67,68,69,74]. In the remaining studies, the intensity ranged from 0.1 to 6.55 mA. The frequency varied between 4 and 30 Hz, and only one study did not report the frequency used [63]. The duration of stimulation varied considerably across studies. Some studies applied stimulation for 20 min [64], 30 min [74], or 1 h [59] for a total of 20 sessions; one study applied stimulation for 45 min daily over 28 days [72]; one study applied stimulation for 30 min for 30 days divided into 15 sessions [66]; one study applied stimulation for 30 min daily over 15 days [69]; and two studies applied stimulation for 30 min across 24 sessions [62,67]. Finally, other studies applied stimulation for 1 session [63], 9 sessions [60], 10 sessions [61,65], 18 sessions [71,73], and 20 sessions [70]. Only one study did not report the duration of stimulation [68].

Most clinical studies have demonstrated positive effects of invasive or non-invasive VNS on outcomes related to stroke. Only one study demonstrated that taVNS showed positive effects on motor function, but not on cognitive function [63] (Table 2).

**Table 2:** Characterization of clinical trials included in the review.

Author/Year	Study Design	Time-Point of Intervention	Outcomes/Results
tcVNS			
Arsava et al. 2022 [68]	Randomized, sham-controlled, open-label, multicenter trial	Hyperacute stroke	Safety and feasibility of nVNS when delivered immediately after confirmed imaging diagnosis of acute stroke, within 6 h of symptom onset. Results: significant.
taVNS			
Li et al. 2022 [64]	Two-group, pragmatic, double-blinded, randomized controlled trial	Subacute stroke	Motor and sensory functions and emotional response. Results: significant.
Wang et al. 2024 [74]	Double-blind, randomized controlled clinical trial	Subacute stroke	Gait of subacute post-stroke individuals. Results: significant.
Liu et al. 2024 [72]	Double-blind, randomized, placebo-controlled trial	Subacute stroke	Level of depression; daily life function; and serum levels of CREB1, BDNF, and 5-HT. Results: significant.
Wang et al. 2022 [66]	Double-blind, prospective, randomized controlled clinical trial	Subacute stroke	Swallowing function in acute stroke patients. Results: significant.
Wu et al. 2020 [69]	Prospective, single-blinded, randomized controlled trial	Subacute stroke	Efficacy and safety of taVNS in the recovery of upper limb motor function. Results: significant.
Wang et al., 2024 [59]	Double-blinded, randomized, controlled pilot trial	Subacute stroke	Upper extremity function. Results: significant.
Zhang et al., 2025 [62]	Prospective randomized controlled clinical trial	Subacute stroke	Central nervous system remodeling and neurological function recovery. Results: significant.
Yan et al., 2025 [67]	Prospective randomized controlled clinical trial	Subacute stroke	Swallowing dysfunction and assessment of serum levels of IL-1β and IL-8. Results: significant.
Capone et al., 2017 [61]	Double-blind, semirandomized, sham-controlled trial	Chronic stroke	Upper limb in chronic stroke. Results: significant.
Chang et al., 2021 [60]	Double-blind, sham-controlled, repeated measures trial	Chronic stroke	Upper limb motor function. Results: significant.
Colombo et al., 2023 [63]	Single session, single-blind, sham-controlled study with a within-subject design	Not specified	Upper limb motor function and cognitive function in individuals with stroke. Results: significant to motor function, non-significant to cognitive function.
TDCSVN			
Kimberley et al., 2018 [70]	Randomized, sham stimulation-controlled, fully blinded trial	Chronic stroke	Safety, feasibility, and efficacy of VNS combined with upper limb rehabilitation. Results: significant.
VNS through rTMS			
Lin et al., 2018 [65]	Sham-controlled, double-blinded parallel study	Chronic stroke	Swallowing function in patients with stroke involving the brainstem. Results: significant.
iVNS			
Dawson et al., 2016 [71]	Randomized open active comparator study with blinded objective end point assessment	Chronic stroke	Upper limb motor function. Results: significant.
Dawson et al., 2021 [73]	Pivotal, randomized, blinded, controlled trial	Chronic stroke	Upper limb motor function. Results: significant.

IL-1β: interleukin-1 beta; IL-8: interleukin-8; taVNS: transcutaneous auricular vagus nerve stimulation; iVNS: invasive vagus nerve stimulation; TDCSVN: transcranial direct current stimulation on the vagus nerve; rTMS: repetitive transcranial magnetic stimulation; VNS: vagus nerve stimulation; BDNF: brain-derived neurotrophic factor; CREB1: cAMP response element binding protein 1; cAMP: cyclic adenosine monophosphate; 5-HT: 5-hydroxytryptamine.

**Table 3:** Clinical characterization of clinical trial participants.

Author/Year	Age of Participants				Gender				Duration of Disease			
tcVNS												
Arsava et al., 2022 [68]	tcVNS 71 ± 14		Sham 71 ± 11		F(17); M(27)				Symptom onset time within 6 h			
taVNS												
Li et al., 2022 [64]	taVNS 69.2 ± 12.3		taVNS sham + Rehab 68.3 ± 12.1		taVNS F(50); M(15)		taVNS sham + Rehab F(47); M(14)		taVNS 10.8 ± 7.7 days		taVNS sham + Rehab 10.4 ± 6.9 days	
Wang et al., 2024 [74]	tDCS 63.16 (5.75)	taVNS 60.82 (6.19)	taVNS + tDCS 61.54 (5.78)	Control 61.94(3.28)	tDCS F(24); M(18)	taVNS F(23); M(21)	taVNS + tDCS F(21); M(22)	Control F(23); M(17)	tDCS 20.05 (4.28) days	taVNS 19.24 (5.83) days	taVNS + tDCS 18.98 (4.56) days	Control 18.89 (5.17) days
Liu et al., 2024 [72]	taVNS 64.3 (10.1)		taVNS sham 62.6 (9.2)		taVNS F(18); M(22)		taVNS sham F(15); M(25)		taVNS 17.3(7.1) days		taVNS sham 18.4(6.8) days	
Wang et al., 2022 [66]	taVNS 60.26 ± 5.65		taVNS sham 58.25 ± 6.86		taVNS F(10); M(9)		taVNS sham F(12); M(8)		taVNS 22.00 ± 3.35 days		taVNS sham 23.70 ± 2.95 days	
Wu et al., 2020 [69]	taVNS 64.50 ± 9.97		taVNS sham 61.82 ± 10.63		taVNS F(5); M(5)		taVNS sham F(3); M(8)		taVNS 36.30 ± 9.23 days		taVNS sham 35.55 ± 6.47 days	
Wang et al., 2024 [59]	VNS 55(11)		VNS sham 57(11)		VNS F(2); M(18)		VNS sham F(5); M(15)		VNS 3.20(2.04) months		VNS sham 4.15(1.60) months	
Zhang et al., 2025 [62]	tVNS + Rehab 62.67 ± 7.34		Rehab 61.59 ± 7.87		tVNS + Rehab F(25); M(38)		Rehab F(25); M(36)		tVNS + Rehab 4.28 ± 1.46 months		Rehab 4.22 ± 1.38 months	
Yan et al., 2025 [67]	TDCSVN ≤60 y: 26 >60 y: 30		Rehab ≤60 y: 28 >60 y: 29		TDCSVN F(18); M(38)		Rehab F(21); M(36)		Within 3 months after the stroke			
Capone et al., 2017 [61]	tVNS 53.71 ± 5.88		tVNS sham 55.60 ± 7.12		tVNS F(3); M(4)		tVNS sham F(2); M(3)		tVNS 93.71 ± 38.81 months		tVNS sham 46.00 ± 21.85 months	
Chang et al., 2021 [60]	59.02 ± 1.98				F(18); M(18)				2.16 ± 0.39 years			
Colombo et al., 2023 [63]	45 to 79				F(4); M(6)				Not specified			
TDCSVN												
Kimberley et al., 2018 [70]	VNS 59.5 ± 7.4		Control VNS 60.0 ± 13.5		VNS F(4); M(4)		Control VNS F(4); M(5)		VNS 18(11–43) months		Control VNS 18(6.3–53) months	
VNS through rTMS												
Lin et al., 2018 [65]	rTMS real 68.5 ± 12.8		rTMS sham 72.9 ± 12.2		rTMS real F(1); M(12)		rTMS sham F(6); M(9)		rTMS real 25.2 ± 42.2 months		rTMS sham 21.6 ± 19.5 months	
iVNS												
Dawson et al., 2016 [71]	VNS + Rehab 57.9 ± 17.2		Rehab 60.7 ± 10.7		VNS + Rehab F(2); M(7)		Rehab F(2); M(9)		VNS + Rehab 1.8 ± 1.0 year		Rehab 1.7 ± 1.3 year	
Dawson et al., 2021 [73]	VNS 59.1 ± 10.2		Control VNS 61.1 ± 9.2		VNS F(34); M(19)		Control VNS F(36); M(19)		VNS 3.1 ± 2.3 years		Control VNS 3.3 ± 2.6 years	

Mean(range). taVNS: transcutaneous auricular vagus nerve stimulation; VNS: vagus nerve stimulation; Rehab: rehabilitation; TDCSVN: transcranial direct current stimulation on the vagus nerve; rTMS: repetitive transcranial magnetic stimulation.

**Table 4:** Parameters of clinical studies.

Author/Year	Intensity	Frequency	Duration
tcVNS			
Arsava et al., 2022 [68]	adjusted (0 to 40)	25 Hz	?
taVNS			
Li et al., 2022 [64]	1.71 ± 0.5 mA	20 Hz	20 min for 20 sessions
Wang et al., 2024 [74]	Adjusted (tDCS: 2.0 mA 30 min/day)	25 Hz	30 min for 20 sessions
Liu et al., 2024 [72]	1.82 ± 0.4 mA	20 Hz	45 min daily for 28 days
Wang et al., 2022 [66]	1.83 ± 0.5 mA	25 Hz	30 min for 30 sessions in 15 days
Wu et al., 2020 [69]	adjusted	20 Hz	30 min daily for 15 days
Wang et al., 2024 [59]	6.55 ± 1.57 mA	25 Hz	1 h for 20 sessions
Zhang et al., 2025 [62]	adjusted	4 and 20 Hz	30 min for 24 sessions
Yan et al., 2025 [67]	adjusted	4 and 20 Hz	30 min for 24 sessions
Capone et al., 2017 [61]	adjusted	20 Hz	10 sessions
Chang et al., 2021 [60]	0.1 to 5.0 mA	30 Hz	9 sessions
Colombo et al., 2023 [63]	0.8 mA	?	1 session
TDCSVN			
Kimberley et al., 2018 [70]	1 mA	20 Hz	20 sessions
VNS through rTMS			
Lin et al., 2018 [65]	adjusted	5 Hz	10 sessions
iVNS			
Dawson et al., 2016 [71]	0.8 mA	30 Hz	18 sessions
Dawson et al., 2021 [73]	0.6 to 1.0 mA	30 Hz	18 sessions

tcVNS: transcutaneous cervical vagus nerve stimulation; taVNS: transcutaneous auricular vagus nerve stimulation, iVNS: invasive vagus nerve stimulation, tDCS: transcranial direct current stimulation, TDCSVN: transcranial direct current stimulation on the vagus nerve, rTMS: repetitive transcranial magnetic stimulation; VNS: vagus nerve stimulation; ?: unclear data.

## 4. Discussion

This review aimed to identify the outcomes evaluated in preclinical and clinical studies on the effects of VNS in post-stroke treatment. Of the 57 studies included, 41 studies are preclinical and 16 are clinical studies, highlighting the efforts of neuroscience to understand the pathophysiological mechanisms of this type of intervention after stroke. The main outcomes of the preclinical studies were the effects of VNS on neuroprotection and motor and cognitive functional recovery.

The most common type of stroke is ischemic, and it is one of the most frequent causes of disability worldwide. Currently, therapeutic approaches involve rapid reperfusion mechanisms; however, rechanneling of blood flow can cause ischemia/reperfusion injury [75]. Stroke is a heterogeneous disease, so animal models are essential tools to mimic these processes in the investigation of pathophysiology and therapeutic approaches [76].

In the present study, the main model of stroke induction in animals was MCAO, representing 73% of the studies. This focal ischemic technique involves the introduction of an intraluminal filament into the external carotid artery and advancing it until it blocks the origin of the middle cerebral artery, producing a more reliable infarction model. The model can be used to establish permanent or transient ischemic stroke, depending on the reperfusion time points. In addition, this approach can demonstrate common pathophysiological features, such as energy failure, elevated intracellular Ca^2+^ level, excitotoxicity, free radical generation, destruction of the blood–brain barrier, inflammation, contribution of glial cells, apoptosis, and necrosis [77].

From the pathophysiological point of view of stroke, the lack of oxygen and glucose during ischemia triggers a series of oxidative, biochemical, and hormonal reactions that culminate in lesions of the microvasculature and rupture of the blood–brain barrier, in addition to triggering secondary inflammatory cells and their respective mechanisms, which can lead to excitotoxicity through reactive oxygen species (ROS), among others [77]. In other words, brain damage due to ischemia triggers a signaling cascade that acts synergistically to induce neuronal death [76]. This highlights the importance of studies that understand both the mechanisms involved in the pathophysiology of stroke and the possible benefits of VNS techniques in this context.

We can observe that 61% of the preclinical studies included here focused their efforts on investigating the use of VNS for neuroprotection in stroke models, with specific outcomes in the activation of α7nAChR in five studies, in the different inflammatory mechanisms in seven studies, and in the volume of cerebral infarction in five studies and pro-angiogenic activity in four studies, placing neuroprotection as an important point of investigation before, during, or after an ischemic episode.

Neuroinflammation after stroke was the main target of studies that investigated the neuroprotective effects of VNS. The literature reports that resident microglia and macrophages in the brain are activated minutes after the onset of ischemia, leading to the production of several pro-inflammatory factors, such as interleukin-1β (IL-1β) and tumor necrosis factor alpha (TNF-α), in addition to promoting excitotoxic injury and ischemia, which may aggravate tissue damage [77]. Seven studies investigated neuroprotection by inflammatory pathways through nuclear transcription-κB (NF-κB), neutralization of USP10, reduction in neuron-derived IL-1β, activation of Cx43, microglial polarization through inhibition of the TLR4 pathway, and M2 microglial polarization by inhibition of the expression of IL-17a, L-PGDS, and by signaling pathways for cell apoptosis and regulation of autophagy, in addition to other studies that investigated neuroinflammation in a secondary manner.

Of the nine studies that investigated the effects of VNS on rehabilitation in animal models, the main outcomes were cognitive recovery; learning recovery, memory, and the mechanisms involved; functional recovery for forelimbs; and neuroplasticity in the corticospinal tract. Although studies were found that evaluated neural mechanisms possibly triggered by VNS, only one study was found that aimed to evaluate neuroplasticity promoted by VNS in animal models after stroke [41].

Regarding clinical studies, only sixteen studies were found and included in this review. We can observe that the outcomes studied were the effects of VNS on the rehabilitation of upper limb motor function; cognitive or sensory rehabilitation and emotional responses; effects of VNS on swallowing function; safety and feasibility of the VNS application; gait in subacute post-stroke; level of depression; daily life function and serum levels of CREB1, BDNF, and 5-HT; and comparison of active VNS paired with rehabilitation versus sham stimulation paired with rehabilitation. There is still a shortage of clinical trials on the effects of VNS in stroke.

Considering that motor functions are the most affected after stroke, including weakness of the upper and lower limbs, ataxias, facial paralysis, language impairments such as dysarthria, and finally, sensory and cognitive deficits [7], we observed that the clinical studies addressed the main impairments observed in individuals after stroke. Upper limb motor function was one of the main outcomes observed. Upper limb dysfunction is highly prevalent in individuals after stroke [78] and is accompanied by reduced use of the affected limb, especially among those with moderate to severe impairments [79]. Few available interventions demonstrate significant effectiveness in improving upper limb function [80]; therefore, it is expected that the scientific community will seek new interventions capable of producing meaningful effects on upper limb motor recovery after stroke. We believe this may justify the larger number of clinical studies investigating the effects of VNS on the upper limb function in this population.

Other impairments affecting individuals after stroke, including deficiencies in lower limb function and trunk control and balance, may increase the risk of falls and, consequently, increase the chances of developing secondary complications, in addition to presenting major dysfunctions when not recovered [81]. Given the impairments resulting from lower limb dysfunction and balance and gait deficits, it becomes important to investigate the effects of VNS on these outcomes to determine whether VNS can produce effects similar or superior to other brain stimulation techniques, considering that only two studies evaluated lower limb-related functions [64,74]. Accompanied by motor impairments, activities of daily living become highly compromised. Future studies may investigate whether the improvement in motor function promoted by VNS translates into better activities of daily living in individuals post-stroke.

Most preclinical studies applied iVNS, followed by taVNS and tcVNS. Conversely, clinical studies applied taVNS, followed by iVNS and tcVNS, in addition to other methodological approaches. The stimulation parameters varied substantially across studies, making it difficult to perform any comparative analysis to suggest the optimal parameters—especially considering that most preclinical and clinical studies demonstrated positive effects regardless of the methodology and parameters applied. There were no studies comparing the effects of left versus right vagus nerve stimulation. The left vagus nerve was preferred, given that the right vagus nerve has a greater influence on the sinoatrial node. This approach may help prevent complications related to the stimulation site. Even though some studies applied VNS at different phases of stroke, no studies compared the effects of VNS on different outcomes across these stroke phases, either in preclinical or clinical research. We observed that most preclinical studies opted to use iVNS, while most clinical studies opted to use taVNS. The use of a non-invasive VNS method in clinical studies helps prevent adverse effects related to device implantation, including infection, lower facial muscle weakness, vocal cord paresis, asystole, and bradycardia [14].

The studies included in this review presented important methodological limitations, including small sample sizes, short follow-up periods, and some inconsistency in the control conditions used. Although the clinical studies showed positive results, these limitations lead to high sample heterogeneity, reduce statistical power, and overlook the long-term effects of the intervention [82,83,84]. Future studies may contribute to a better understanding of the effects of VNS, especially in the long term, on outcomes related to stroke through clinical trials with higher methodological quality. Overall, this review contributes to the current knowledge on the evidence supporting the use of VNS to treat important outcomes related to stroke.

## 5. Conclusions

We conclude that the most studied outcomes of VNS after stroke were performed in animal models and were based on neuroprotection. Clinical trials that evaluate the efficacy of this technique used for rehabilitation, and its comparison or combination with other techniques, remain scarce. We can also observe other outcomes that are still little explored, including lower limb function, trunk control, balance, and gait, opening room for potential future investigations related to outcomes that directly impact the recovery of individuals after stroke. Future studies will be needed to investigate optimized parameters for the treatment of stroke-related outcomes, given the high variability in stimulation parameters observed in the current literature, which may improve the reproducibility of the intervention by clinicians. Finally, studies with longer follow-up periods may demonstrate the long-term effects of VNS in individuals after stroke.

## 6. Limitations

Searches of the gray literature, as conducted in other scoping reviews, were not performed, although there are recommendations to do so. Only published studies were included in the review, assuming a certain level of methodological rigor among the included studies. This indicates that this review may not have included emerging or unpublished studies.

## Data Availability

All data generated or analyzed during this study are included in this published article. No primary data were collected, as this study is based on previously published literature.
